# Constructing the Landscape Ecological Security Pattern in the Dawen River Basin in China: A Framework Based on the Circuit Principle

**DOI:** 10.3390/ijerph20065181

**Published:** 2023-03-15

**Authors:** Jianchun Li, Rong Shan, Wenhua Yuan

**Affiliations:** 1 Business School, Shandong Normal University, Jinan 250014, China; 2 School of Economics and Management, China University of Petroleum, Qingdao 266580, China

**Keywords:** landscape ecological security pattern, circuit principle, ecological corridor, collaborative development

## Abstract

With the rapid development of urbanization, land cover, and land use patterns have greatly changed in China, which has damaged the landscape structure, affected the energy balance and material flow within the system, and reduced the value of ecosystem services. The construction of landscape ecological security patterns could promote species exchange between biological groups and increase material and energy exchange between landscape elements. Few studies have focused on the randomness of species to migration path, thus failing to objectively reflect the process of species migration and diffusion. Therefore, circuit theory was used in this study to better match the random selection of migration paths by species. In this paper, 14 typical mammal species from the Dawen River basin of the lower Yellow River in China were selected as examples, and the conclusions were as follows: (1) The ecological sources of the Dawen River basin were 49, with forest land and lakes as the main sources, and they were crucial to the stability of the regional ecological security pattern. A total of 128 ecological corridors were identified, among which 83 were key corridors and the rest were potential corridors. The key corridors throughout the whole region need priority protection and can be used as a core area for the observation and monitoring of natural resources. (2) Based on the circuit principle, 32 pinch points and 21 barrier points were identified, indicating that regional habitat connectivity must be further improved. (3) Four types of zones were determined, and optimization measures were proposed. (4) On the basis of conceptual protection, the ecological protection network of the Dawen River basin was built to enhance ecological resilience. The landscape ecological security pattern of the Dawen River basin was constructed from the three levels of points, corridors, and areas. Based on the concept of regional ecological security, a resource optimization strategy for ecological security patterns was proposed, which is significant for maintaining the integrity of watershed ecosystems.

## 1. Introduction

As an important developing country, China’s rapid urban expansion and industrialization processes have caused serious damage to the ecosystem in many regions [1]. The increasingly frequent ecological problems, such as floods and drought, soil loss, and sharp biodiversity declines, have gradually awakened people’s general awareness of ecological security [2]. Under the strategic positioning of “ecological priority and green development” in China, identifying landscape ecological security patterns is an important issue to achieve regional sustainable development. The establishment of landscape ecological security patterns can not only provide basic regional protection for necessary ecosystem services and a healthy living environment but also effectively guide regional development and construction [3,4,5]. 

In order to identify landscape ecological security patterns, landscape connectivity was often used [6,7]. By understanding the connectivity patterns within a landscape, it is possible to identify areas that are important for maintaining ecological functioning and to develop strategies for conserving these areas. In addition, spatial analysis tools such as GIS or remote sensing are also commonly used, which could identify areas of high ecological value, such as wetlands, forests, or grasslands [8,9,10]. These methods emphasize the protection of regional ecological security from different perspectives, but how to systematically build an ecological pattern is still the focus of research. Currently, the paradigm of “ecological source identification—Resistance surface construction—Corridor extraction—Security pattern construction” is commonly used to analyze landscape ecological security patterns [11,12,13].

Under the paradigm, scholars have adopted different methods to study landscape ecological security patterns. Some scholars identify the source areas by evaluating ecological sensitivity, ecosystem services, and habitat quality [14,15]. Some scholars also use slope, elevation, and other factors to construct the resistance surface, and then further modify it with night light data [16,17]. The traditional methods to identify ecological corridors include the least cost path [1,18], the spatial pattern index [19], and the ant colony algorithm [20] based on animal population diffusion. These methods regard ecological corridors as the optimal path of species migration and diffusion and ignoring the randomness of species selection migration paths; additionally, different species usually choose different migration corridors.

Hence, circuit theory was introduced by McRae [21], which is a tool used to test assumptions about how landscape connectivity and landscape characteristics promote or hinder animal movement and how they affect local wildlife population diffusion and gene flow [22]. According to the circuit theory model, the current flowing between any pair of nodes is equivalent to the number of times the simulated individual moves along that path [23]. Thus, based on the current density between nodes, potentially important paths that affect landscape connectivity can be identified [24,25]. The alternative method, the least-cost path, is often used to identify possible corridors or diffusion paths, often identifying one least-cost path [22]. The circuit theory model allows the identification of multiple candidate movement paths, which improves the least-cost model to some extent. In the absence of sufficient motor data obtained during birth transmission, circuit theory can be applied afterward to identify potential transmission corridors, or routes that clandestine animals may use depending on their habitat. Scholars have proved that circuit theory is a useful tool by using a landscape probability model, landscape resistance scenario setting, and other methods, which can identify potential mammalian motion paths in the case of limited high-resolution motion data [26,27,28].

Dawen River basin is the largest branch basin of the lower Yellow River in China. The basin integrates various natural landscapes of mountains, waters, and lakes. The north and east are mountains, including Mount Tai, Mount Lu, and Mount Meng. The west and south are hills and plains. There are abundant animals in the Dawen River basin, among which mammals include Rodentia (such as Chinese hamster, Cricetulus triton, Black-threaded rat, etc.), Erinaceomorpha (such as Stabbs), Carnivora (such as wolves, Grass fox, badgers, and weasels), Chiroptera (such as Hairy chrysanthemum bats), and Lagomorpha (such as grass rabbits). After decades of development, the Dawen River basin has achieved remarkable economic development, but it also has a great impact on the local ecological environment, leading to the loss of mammalian habitats and increasing threats of fragmentation. Thus, the aims of this study were to (1) determine the ecological source according to the habitat quality of the Dawen River basin; (2) establish the ecological resistance surface using different types of mammals; (3) identify important corridors and key points according to the circuit theoretical model; and (4) develop ecological regulation zoning for the river basin.

## 2. Study Area and Data Sources

### 2.1. Study Area

**Figure 1:**
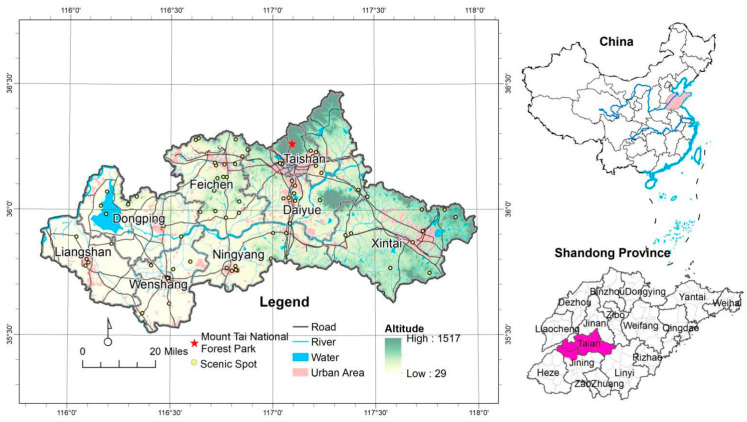
Location and overview of the Dawen River basin.

The Dawen River is located in the middle of Shandong Province, which is the largest and last tributary of the lower reaches of the Yellow River (Figure 1), which flows through eight counties and cities, including Liangshan County (LS), Dongping County (DP), Wenshang County (WS), Feicheng City (FC), Ningyang County (NY), Taishan District (TS), Daiyue District (DY), and Xintai City (XT). Dawen River basin is located between 115°51′53″–117°59′54″ E and 35°30′47″–36°28′34″ N. The total length of the Dawen River is 209 km, the area is 8634 km^2^, and the average elevation is 213 m.

The Dawen River basin belongs to semi-humid and semiarid areas based on rainfall. The rainfall in the river basin is abundant, and the rainfall is relatively concentrated in the summer from June to September. The total rainfall can account for 75% of that for the whole year, and the area is prone to flood disasters (http://www.taian.gov.cn/, accessed on 18 November 2022). The early intensity of human activity in the Dawen River basin was weak, and there was a subtle low-level balance between people and ecology. However, in recent years, urbanization and industrialization in the Dawen River basin have developed rapidly. In 2022, the total population of the region was 7.378 million, the urbanization rate was about 50%, and the GDP was CNY 217.8 billion with an average growth rate of 11.19% over the past 20 years. With the acceleration of urban construction, the long-term interaction balance between human beings and the environment has been broken. Serious water and soil loss in the Dawen River basin, serious industrial wastewater pollution, and serious biodiversity damage result in unstable ecological and environmental quality, weak links in flood control and disaster reduction, prominent shortcomings in water resource utilization, and an arduous task for economic transformation and development. 

### 2.2. Data Sources

This paper focuses on three aspects of geographic data source selection, data processing, and field research to obtain the data and information needed for this study.

In terms of geographic data selection, settlements, roads, and water systems in the study area were obtained from the National Basic Geographic Database (1:1,000,000) (http://www.ngcc.cn/ngcc/ (accessed on 1 May 2022)). The elevation data were obtained from the Geospatial Data Cloud Platform (https://www.gscloud.cn/ (accessed on1 May 2022)). The land use coverage data were obtained from Global Geo-information Public Product (http://globeland30.org/ (accessed on 1 May 2022)). The multi-period historical high-resolution remote sensing images were obtained from the BIGEMAP platform (http://www.bigemap.com/ (accessed on 1 May 2022)).

In terms of the data processing process, the reference time of the study data was unified to 2020, and the available historical data were also incorporated into the study database. According to the spatial processing method of the GIS platform, all data were unified at a spatial resolution raster of 30 m × 30 m [29]. The DEM was used to calculate the slope and topographic relief of the Dawen River. The distribution of important nature conservation sites in the region was determined by identifying specific types of land use, including local names and areas. Integrated remote sensing images and watershed land use change survey database provided a reference for restoration work. In response to the regional economic development status, natural and historical and cultural elements are integrated to build a basic database of landscape ecological security.

In terms of the field research and study, for the Dawen River flowing through seven counties (cities) in Shandong Province from east to west, the research team conducted several studies on the status of natural resources and social development in this region between October 2019 and June 2020 using the Taishan National Forest Park as the starting point and the area around the river as a sample zone. A case study of typical regional studies was also launched to obtain the following data and information. Firstly, the distribution of watershed roads, industrial parks, and towns in the region was clarified, and the scope of their impact on biological migration was assessed. Secondly, the spatial distribution of resources such as forest, grassland, water surface, and arable land was further verified and solved using point observations. Thirdly, the characteristics of key biological species in the watershed were investigated to obtain their number and distribution status.

## 3. Analytical Framework and Methods

### 3.1. Analytical Framework

Ecological sources are large ecological plaques with the main radiation function of the region. For the determination of the source area, most studies directly select natural reserves, scenic spots, and other habitat patches that are important to regional ecological security [30,31]. A resistance surface refers to the obstacles encountered by organisms in the process of migration, which is mainly obtained with the assignment of land use types [32]. An ecological corridor shows the ecological flow, ecological process, and ecological function transfer of a region [33]. The concept of landscape ecological security patterns refers to the patterns in landscape structure and function that are related to the stability and resilience of ecosystems within a landscape [34]. The elements of the patterns can include land use, land cover, vegetation, topography, and hydrology, among other variables. By understanding the relationships between these elements, it is possible to identify key drivers of landscape change and to develop strategies for promoting landscape ecological security [35].

**Figure 2:**
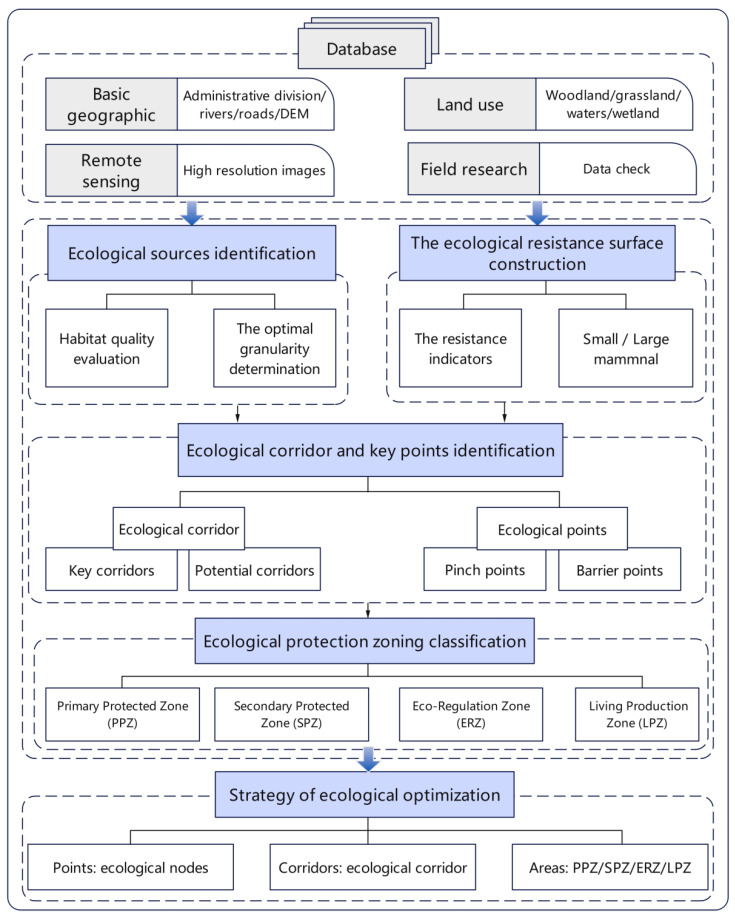
The research framework.

The research framework of this paper is shown in Figure 2. First, the database of landscape ecological security pattern evaluation was established. We collected a basic database for this research, which included remote sensing, urban planning, roads, natural geography, and land use data. Second, we determined the ecological source based on the principles of landscape ecology and constructed an ecological resistance surface combined with regional land use type, geomorphic factors, and road factors. Third, the ecological corridors were constructed, and key points were identified using circuit theory, identifying the key and potential corridors, as well as the pinch and barrier points. Fourth, based on a comprehensive consideration of habitat quality, the improvement coefficient, and the actual situation of the basin, the ecological security pattern was established, and the necessity of regional ecological protection was divided into four types, including the Primary Protected Zone (PPZ), Secondary Protected Zone (SPZ), Eco-Regulation Zone (ERZ), and Living Production Zone (LPZ). Finally, according to the ecological nodes, ecological corridor, and the three different areas, targeted restoration and control strategies are proposed from the aspects of point, corridor, and area to reconstruct the ecological security pattern of the basin.

### 3.2. Methods

#### 3.2.1. Ecological Sources Identification

1. Habitat Quality Evaluation

Habitat quality refers to the ability to reproduce and the presence of quantitative ecosystems based on survival resource availability to provide survival conditions suitable for individuals and populations [36]. It depends on the proximity of humans to the land and the intensity of land use. A high-quality habitat is relatively complete with both structure and function within the historical dynamic range. The degradation of habitat quality is regarded as the result of the increase in the intensity of nearby land use. 

The habitat quality model is one of the important modules of the InVEST model, which evaluates habitat quality from a biodiversity perspective, including external threat intensity and the sensitivity of different land use types to threat sources [37,38]. The model believes that habitat quality is high in areas with many biological species and good natural environmental conditions and is less affected by threatened sources. In the application principle, the model is evaluated based on a grid, each endowed with a type of land use, and for each evaluated landscape, the model requires a density map (or intensity) of the threat factors on the entire landscape. Based on the above factors, threat data layers are used to assess the degree of habitat quality degradation for different land use types.

The threat source has certain attributes and has an impact on the habitat quality of the plot, mainly including the threat ability, the sensitivity, and the distance between the plot unit and the threat source. The first attribute is the threat ability, which refers to the range of the habitat impact of each threat layer. The stronger the threat capability is, the higher its relative impact score, and the more destructive it is to the habitat than the other threat types. The second attribute is sensitivity. The relative sensitivity of each habitat type may be different for each source of threat. The stronger the sensitivity, the lower the anti-interference ability of the land use type unit, and the quality of the habitat plate deteriorates. The third attribute is the distance between the parcel unit and the source of the threat. Typically, the degree of the threat decreases exponentially or linearly as the grid distance from the threat source increases, and thus, the grid units nearest to the threat will be greatly affected.

Habitat degradation results from external threats, so habitat quality is evaluated based on the strength and sensitivity of the habitat to the threat source. The habitat quality index is calculated as follows:

(1) $$Q_{xj}=H_{j}\left[1-(\frac{D_{xj}^{z}}{D_{xj}^{z}+K^{z}})\right]$$

where *Q_xj_* denotes the habitat quality index of grid *x* on land use type *j*; *D_xj_* is the threat level of *x* grids on land use type *j*; *H_j_* is the habitat suitability; and *k* and *z* are scaling parameters that are constants. The value of *Q_xj_* reflects the habitat quality and the fragmentation degree of the land use type under the influence of the threat sources. Plots with high scores show stable ecosystem structure and good habitat quality; in contrast, plots with low scores have poor interference resistance, and the ecological environment is prone to damage.

According to the InVEST Guide (https://naturalcapitalproject.stanford.edu/ (accessed on 2 May 2022)) and the natural conditions of the Dawen River basin, the habitat types in this paper include woodland, grassland, and waters, and the stress factors include rural settlements, mining land, roads, railways, and rural roads. Then, the digital elevation model (DEM) is used to obtain the habitat quality of the river basin.

The ecological source is an ecological site with high habitat quality and high biological species and biomass [39]. Most studies have directly selected grassland, woodland, and water bodies as natural ecological spaces and then combined the spatial distribution and area size of related ecological sites to screen ecological sources. This method requires a small amount of data and is easy to process, but at the same time, the default ecological site properties are fixed and unchanging, and the ecological site properties caused by the surrounding environment change dynamically. In this study, the ecological sources identification method based on habitat quality analysis was chosen to identify ecological sources with better habitat quality and higher species habitat suitability as ecological sources to better fit the biological conservation needs. Using the InVEST model habitat quality analysis module, grassland, woodland, mudflats, and water bodies were considered as habitats, paddy fields and drylands as semi-habitats, and other types of sites were considered as non-habitats. Urban areas, rural settlements, and industrial and mining sites were selected as threat sources, and the impact distance, weight, and sensitivity of different land cover types were determined by referring to relevant studies and combining expert opinions, and the calculation results were classified into five categories using the natural breakpoint method to finally obtain habitat quality.

2. The optimal granularity determination based on the landscape pattern index

A landscape is a region of spatial heterogeneity consisting of interacting patches or ecosystems, repeated in similar forms. Changes in landscape patterns will affect ecosystems and can be directly reflected in landscape structure and composition. Different landscape types play different roles in protecting species, maintaining regional ecological stability, maintaining biodiversity, and improving the overall structure and function, and their anti-interference ability to the outside world differs.

To determine the research scale, this paper analyzed the connectivity of the research objects under different granularity levels using the granularity backstepping method to determine the selection criteria for ecological sources according to the optimal granularity [40]. Based on the landscape type of the preliminary regional land use landscape, this paper selected six indicators from the perspective of integrity and connectivity, including quantity, shape, fragmentation, agglomeration, and proximity, to determine the appropriate granularity according to the change in overall connectivity [41]. This paper calculated the number of patches (NP), landscape division index (DIVISION), landscape shape index (LSI), Shannon’s diversity index (SHDI), contagion index (CONTAG), and Splitting index (SPLIT) at each grain size level to explore the change characteristics in the overall landscape morphology with the scale transformation (see Table 1).

To determine the optimal granularity level, this paper amplified the granularity with a 30 m grid as the minimum resolution and 30 m as the step length. According to the regularity of the granularity effect of the landscape index, the optimal granularity of the landscape ecological security pattern was analyzed. Nearby ecological patches were constantly merged with increased granularity, and small patches were removed to form landscape components with better connectivity. Combined with the optimal granularity level, the habitat quality was divided into 5 levels using the natural breakpoint method, and the highest level of habitat was selected and systematically screened combined with the division of natural protection sites and forest parks in the river basin.

#### 3.2.2. The Ecological Resistance Surface Construction

Species need to overcome resistance to move in space. The resistance surface based on the principles of ecology and biology is the basic element for building a corridor, and the corridor plays an important role in solving regional species movement. The traditional method for building resistance surfaces mainly simulates ecological resistance based on patch landscape types, which include only dominant resistance [42]. In this paper, the terrestrial mammal species in the Dawen River basin were considered to construct the resistance surface. The mammals in the Dawen River basin belong to Palaearctic and North China, and there are 14 mammal species considered in this study, belonging to 5 orders and 8 families. According to the relevant research [21], mammals were divided into small animals (<300 g) and large animals (>300 g), and resistance values were set respectively. The detailed classification and alias of 14 typical mammal species are shown in Table 2.

Indicators representing the geomorphological conditions and road resistance indicators were selected to build a comprehensive ecological resistance surface, and the factors and weights were set mainly by referring to existing studies [37,42,43]. The setting of resistance values for small and large animals mainly followed the following principles according to the relevant literature: since small animals tend to perceive landscape fragmentation at a finer scale [44,45], smaller barriers can hinder the diffusion movement of some species [43,46], and the resistance values for small animals are generally higher than those for large animals when setting resistance values. However, because large animals are more likely to be exposed during movement on unobstructed objects such as grasslands and artificial surfaces, the resistance value for large animals is greater than that for small animals in these cases (see Table 3).

In this paper, the minimum cumulative resistance (*MCR*) model using relative resistance factors of different hierarchical sources was considered to calculate the resistance values. The ecological resistant surface can be calculated as follows:

(2) $$MCR=MAX\left(\overset{i=m}{\underset{j=n}{\sum }}W_{ij}R_{Small},\overset{i=m}{\underset{j=n}{\sum }}W_{ij}R_{Big}\right)$$

where *MCR* is the maximum ecological cumulative resistance surface of urban expansion; *MAX* indicates that a landscape unit takes the maximum cumulative resistance to different sources; *W_ij_* is the space distance from source *j* to grid *I*; *R_Small_* is the small mammal resistance coefficient of grid *i* in the process of movement; and *R_Big_* is the big mammal resistance coefficient of grid *i* in the process of movement where the higher the grade of urban land is, the stronger the expansion capacity and the smaller the relative resistance factor.

#### 3.2.3. Ecological Corridor Construction and Key Points Identification

1. Ecological Corridor Construction

McRae first proposed the isolation by resistance (IBR) model, which was used to predict the genetic characteristics of species in complex landscapes [47]. This model was gradually used to predict the migration and diffusion process of biological populations, forming the circuit principle. The circuit principle treats individual species or gene streams as electrons, using the concept of alternative landscape resistance surfaces (resistance map) and treating better-quality plots as nodes (node) of alternative ecological sources [48,49]. The higher the habitat quality, the more conducive it is to species migration and the greater the conductivity. Therefore, the connectivity and the number of potential paths between two nodes can be reflected using the current voltage, which represents the number and probability of species migration, and the voltage represents the difference between the patches. Linkage Mapper 2.0 for Arcgis and three of its basic components (Build Network and Map, Pinchpoint Mapper, and Barrier Mapper) were used to run the model. 

The basic data of the study area was determined, including the ecological source and the construction of the resistance surface. Ecological Corridor Construction was calculated as follows. First, based on the Build Network and Map tool, the cost-weighted distance (CWD) for all pixels to the source on the integrated resistance surface was calculated. Second, the cumulative movement cost path between the sources was calculated after stacking the CWD gate with the source. Finally, the minimum cost distance was composed of the least cost distance (LCD), and the corresponding path was the minimum cost path, i.e., the corridor. Among them, the corridor was divided into key and potential corridors according to the default parameters of the model, and the potential corridor can be protected as the backup resource of the key corridor.

2. Key points identification

After identifying the ecological corridor, using the Pinchpoint Mapper tool, different ecological nodes (source places) and input currents were connected to other nodes for iterative calculation. The node that all current (species) must pass is the “pinch point”, which is the area with the largest movement density of species, and key protection is required. Additionally, using the Barrier Mapper tool, the search radius was set to 500 m, and the search was performed using the mobile window method (diameter of *D*). The improvement coefficient is represented by the size of the connectivity recovery value in the unit distance. The greater the improvement coefficient, the greater the landscape connectivity enhancement value is after this region is removed, thus identifying the birth region with the greatest impact on connectivity. The formula is as follows:

(3) $$\Delta LCD=LCD_{0}-LCD_{1}$$

(4) IS=ΔLCD/D

where ∆*LCD* is the least cost distance difference, *LCD*_0_ is the least cost distance, *LCD*_1_ is the least cost distance value after obstacle point removal, and *IS* is the improvement coefficient. The first 10% of the high improvement coefficient is the main obstacle area, measured using the GIS platform.

#### 3.2.4. Ecological Protection Zoning Classification

After identifying key corridors, potential corridors, pinch points, and barrier points using circuit theory, the study area is further divided into four types, including PPZ, SPZ, ERZ, and LPZ, according to the requirements of comprehensive protection requirements (see Table 4). 

The classification plays an important role in guiding regional development under the pattern of ecological security [50]. The Ecological protection Zoning is determined with the following steps: first, the key points and corridors of ecological security identified in the previous article are the basis; and second, the conversion rules are determined by combining the field survey, expert experience, and the relevant literature, and the spatial analysis method is adopted (www.mee.gov.cn/ywgz/fgbz/xzfg/201805/t20180516_440442.shtml/ (accessed on 22 May 2022)). As shown in Table 4, the PPZ includes three key elements: pinch points, ecological corridors, and key ecological sources, as well as buffer zones based on spatial proximity rules. The ERZ mainly includes obstacle points and the farmland and villages within the buffer zone. In addition, it also includes areas with an improvement coefficient (*IS*) greater than 10% found using current intensity analysis in the whole region, which is mainly extracted from farmland and villages from the perspective of the land use type. The SPZ includes potential ecological corridors, ecological sources, and some of the habitat quality, as well as the buffer zone.

## 4. Results

### 4.1. Ecological Sources

**Figure 3:**
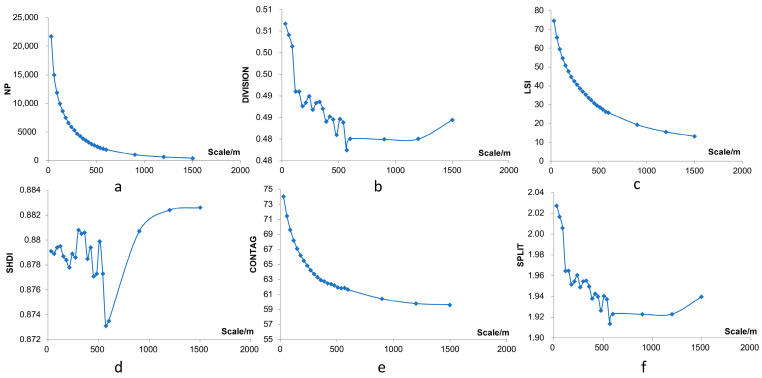
Ecological landscape connectivity with different granularity. (**a**) Number of patches. (**b**) Landscape division index. (**c**) Landscape shape index. (**d**) Shannon’s diversity index. (**e**) Contagion index. (**f**) Splitting index.

For the natural landscape conservation of the study area, the choice of governance scale selection is one of the key issues. Fragstats can evaluate the quantitative parameters of landscape in the study area using inputting images (landscape). We put the land use patches of the study area into Fragstats for simulation and adjusted the granularity of the landscape. To determine the optimal study scale, based on the ecological source, different particle size grid plots were generated from 30 m to 1500 m using the landscape pattern index at different granularity levels calculated with Fragstats (Figure 3). The number of patches and the landscape division index decreased with granularity, and the composition number stabilized after 500 m. For patch cohesion, as the granularity increased slightly, the condensation size decreased sharply after more than 500 m, indicating a strong connectivity for the landscape granularity level of 500 m, which was also applicable to other indexes. As the key point of each landscape index, 500 m can be used as an appropriate reference for the size selection of ecological sources. Thus, 500 m is used as a basis to select the whole ecological study scale, such as the selection of buffer zone classification, spatial data synthesis, zoning the minimum area of the map spot, etc. Due to the limited radiation range of fine fragment patches, patches with habitat areas greater than 2 km^2^ were selected as the ecological source in combination with relevant references.

**Figure 4:**
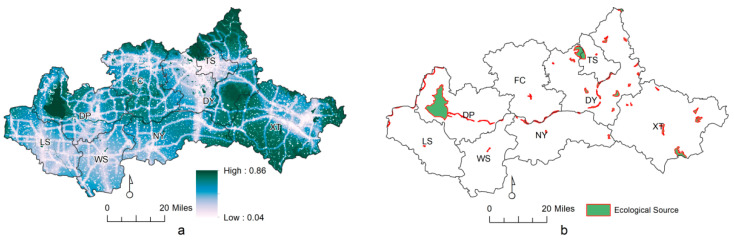
Habitat quality identification and selection of final ecological sources. (**a**) Habitat quality. (**b**) Ecological sources.

The calculated habitat quality of the Dawen River basin is shown in Figure 4. The average value was 0.1967, and the area above the average value accounted for 57.95%. The overall habitat quality of the Dawen River basin was at a low level. The value area was concentrated in the southeast and northwest of the research area, with a large area, and was connected to pieces that were mainly located in TS, DP, DY, and XT. The land use types were mainly woodland and grassland. The low-value area was concentrated in the central and southern urban land, with small areas, and mainly located in FC and NY, accounting for 8.89% of the total area.

This study identified 49 source plots (Figure 4), including its National Forest Park, Dongpinghu, Baifang, and Mount Tai, with an area of 262.25 km^2^, accounting for 2.73% of the total area (9602.68 km^2^). These areas included woodlands, wetlands, and water areas, among which water bodies were the main ecological source type, accounting for 76.66% of the total source area. DP has the largest ecological source area of 139.91 km^2^. For the Dawen River basin, the relatively typical ecological land types that can form are wetlands and water bodies, which are currently less affected by human beings and can form a relatively typical protection area.

### 4.2. The Ecological Resistance Surface

**Figure 5:**
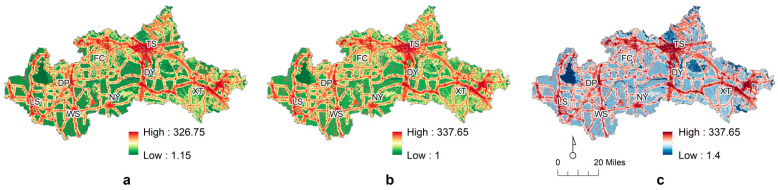
The construction process for the ecological resistance surface in the Dawen River basin. (**a**) Small mammal ecological resistance surface. (**b**) Big mammal ecological resistance surface. (**c**) Maximum integrated resistance value surface.

Based on the data in Table 2, the resistance value was measured, and then the comprehensive resistance surface was constructed (Figure 5). The area with a large comprehensive resistance value was basically located in the urban area with intensive manual activities and around the roads. Although farmland has better-growing vegetation, due to the influence of human agricultural activities and artificial control around it, especially the highly intensive utilization mode, the resistance value directly increased. The small mammal ecological maximum resistance of the entire greater basin was 326.75, the minimum resistance was 1.15, and the average resistance was 90.49. The big mammal ecological maximum resistance of the entire greater basin was 337.65, the minimum resistance was 1.00, and the average resistance was 82.21. The comparison revealed a difference between the combined resistance surfaces of small and big mammals, but the difference between the average resistance values was relatively weak. The maximum value of integrated resistance was 337.65, the minimum value was 1.40, and the mean value was 96.40. The resistance value distribution was relatively fragmented, similar to the basic resistance surface of the land use type. After the superposition of the factor resistance data, the internal resistance value will vary; specifically, except for the high-value area that is concentrated in the central city and northwest, the rest are scattered. 

### 4.3. Ecological Corridor and Key Points

#### 4.3.1. Ecological Corridor Identification

**Figure 6:**
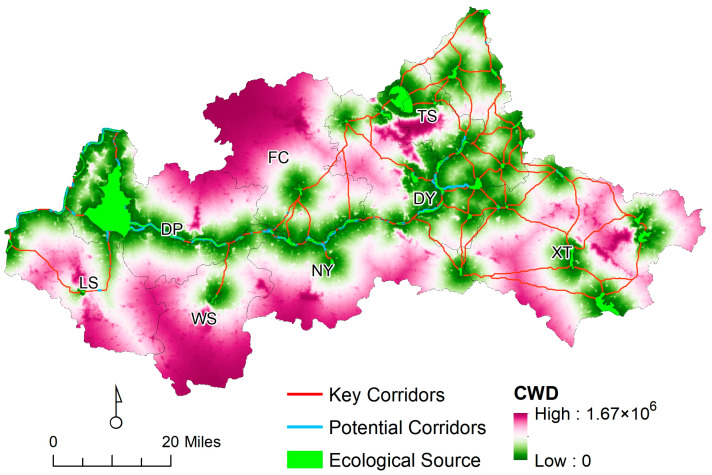
The corridors in the Dawen River basin.

Ecological corridors are the basic architecture for maintaining regional ecological security by connecting different sources, increasing regional landscape connectivity, and maximizing ecological benefits. According to the calculation results using circuit theory, there are 128 ecological corridors identified in the Dawen River basin. Among them, there are 83 key corridors with a length of 953.21 km, and the rest are potential corridors with a length 388.63 km (see Figure 6). The overlap of some critical and potential corridors in specific sections was excluded, totaling 1341.84 km.

#### 4.3.2. Key Point Identification

**Figure 7:**
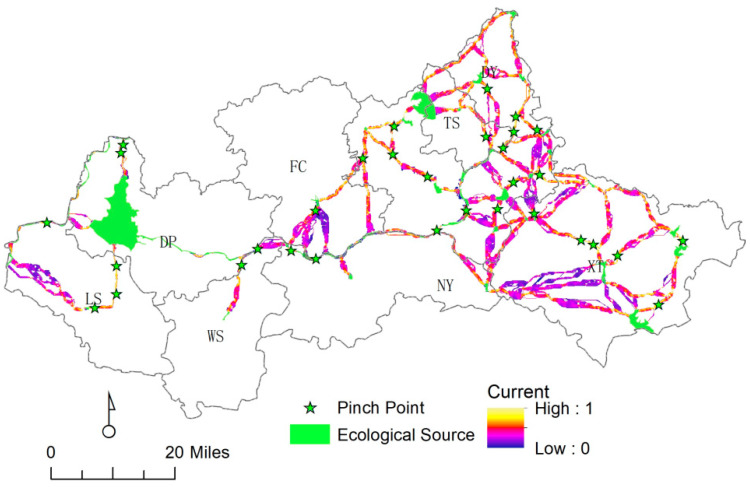
Current strength and pinch point identification for the Dawen River basin.

Based on the circuit principle, 32 pinch points were identified, most of which were in the ecological conservation section, indicating that protected pinch point areas could significantly improve landscape connectivity (see Figure 7). The land use types of pinch points are mainly water areas. Specifically, there were 2 in DY, 2 in FC, 13 in DY, and 2 in TS (see Table 5).

**Figure 8:**
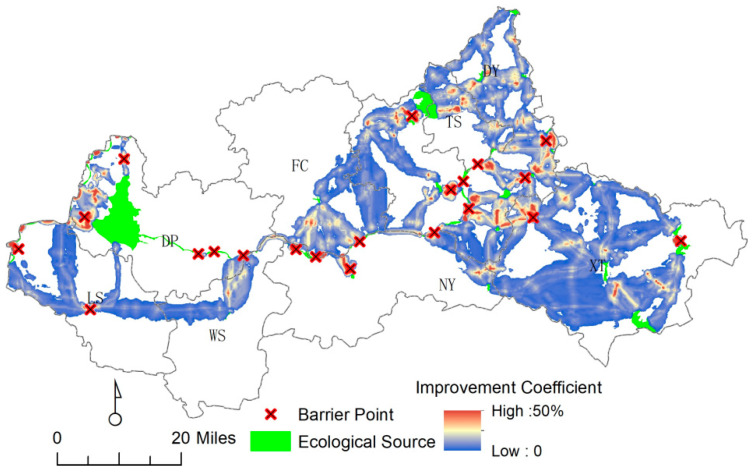
Current strength and barrier point identification for the Dawen River basin.

In addition, 21 barrier points were identified, mainly including construction land, roads, and rural settlements (see Figure 8). Specifically, there were five in DP, two in FC, seven in DY, and one in TS. Since such areas of human activities are often relatively dense, most of the spatial distribution was mostly located at the connection of ecological sources and ecological corridors, which were the key positions of communication. Therefore, there is still much room for improvement in the Dawen River basin regional landscape connectivity.

### 4.4. Ecological Protection Zoning

The ecological security structure in the Dawen River basin was established based on natural resources observation and survey data. The area was classified into four regions according to habitat quality and regional comprehensive protection requirements (Table 6). The PPZ includes the ecological source area with the highest habitat quality and key corridors. The scope of improvement areas was determined using Barrier Mapper tools, and it was divided into two levels with natural breakpoints based on the improvement coefficient. The higher improvement coefficient was the SPZ (0.05 < IS ≤ 0.15), which was the key area of ecological recovery; the lower was the ERZ (0.00 < IS ≤ 0.05); and the rest was the LPZ.

**Figure 9:**
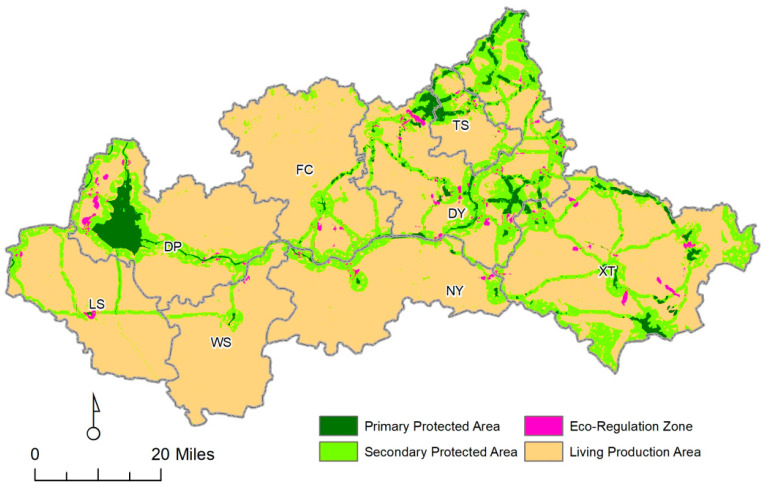
Ecological protection and restoration zones of the Dawen River basin.

The PPZ was 42,175.81 km^2^, accounting for 4.40% of the total area, mainly composed of large areas of high-level habitats. The land type is mainly woodland, wetland and water, and a small amount is garden land. The ecological corridor of this section focuses on wetland ecological protection. High vegetation coverage and rich biodiversity are important for the conservation and maintenance of biological species and their habitat, which can give priority to endangered development through their own powerful regulatory function. The ERZ improvement area of 9164.25 km^2^ was 0.95% of the total area, mainly sandwiched by barrier points and ecological corridor composition. The ecological corridor of the development area highlights the construction of the urban wetland park and the urban Dawen River landscape belt. The core ecological source current intensity of “one mountain and one lake” was the strongest, with the species movement density (current intensity) gradually enhanced from eastern DP to Taishant in the northeast (Figure 9). Ecological sources, ecological nodes (pinch points and barrier points), ecological corridors, and ecological zones together constitute the ecological network of the Dawen River basin.

## 5. Discussion

### 5.1. The Key Points in Dawen River Basin

According to the field survey and relevant references, the status quo and existing problems of key points are analyzed in this section (Table 7). In view of the river pinch points, restoring the river buffer zone, planting ecological protective forests along the coastline, strengthening wetland construction along the river, and improving wetland water conservation is important. Attention should be paid to river pollution control, the control of industrial pollution sources along the line should be strengthened, and the construction of urban sewage centralized treatment facilities should be sped up. Efficient and green pollution-free fertilizer should be promoted, and the development mode of combining industry, sightseeing, ecological functions, and agriculture should be implemented. In view of the woodland pinch points, attention should be given to returning farmland to forest and grassland and strengthening the construction of woodland on both sides of the river. Permanent basic farmland, towns, villages, and mining rights that have been allocated into the core reserve areas are gradually withdrawn in a gradual and orderly manner. Having been transferred into the general control area, the gradual exit in an orderly manner will affect the ecological function.

For the barrier points, we found that most of them cannot be directly removed because of some traffic arteries and areas with important living functions. In this case, biological channels should be built in obstacle areas on both sides of important roads, and warning signs should be set up to ensure the smooth movement of animals. The landscape design on both sides of the expressway should be performed to reduce the slope protection damage to vegetation, and planting greening should also be adopted in the central isolation belt to maintain the diversity in landscape diversity. By enriching planting varieties, local advantageous tree species can be chosen to avoid reducing biodiversity due to single planting and to improve the quality of the forest network. The use of chemical fertilizers and pesticides should be reduced under the condition of ensuring the proper yield and stabilizing the soil properties. 

### 5.2. The Ecological Corridor in the Dawen River Basin

The ecological corridor runs east to west throughout the study area. The key corridors are centered on the Dawen River and extend to both sides. The CWD value of the key corridors is lower, and the connectivity between the corresponding ecological sources is higher. The key corridors are the arteries for improving regional connectivity and are the key ecological corridors to be protected when optimizing the ecological safety pattern. The results show that potential corridors have relatively low CWD values and high connectivity between their corresponding ecological sources. It can be hypothesized that potential corridors play a key role in improving regional connectivity under ideal conditions and are ecological corridors that need to be focused on in the optimization of safety patterns.

The ecological corridors of the Dawen River basin can be divided into two main groups: the east side and the west side, with the FC–NY boundary as the midpoint, the west side is dominated by the key ecological corridor along the east–west direction of the Dawen River, mainly composed of long corridors, with a corridor length of 10 km or more. On the east side, the corridor distribution is gradually complicated, and it is surrounded by a network in the whole eastern region, mainly composed of short corridors, with the length of corridors within 10 km.

Among all the corridors, the ecological sources in the northern and central parts were mainly connected by short corridors with lengths within 10 km, showing fine fragmentation characteristics, accounting for 32.80% of the total key corridors, and the regional corridors overlapped, which is a manifestation of frequent species movement. The northwest and southeast were far separated from the ecological sources. From the northwest to southeast, it was gradually complex and gridded, and the connectivity was gradually enhanced, forming a systematic ecological security pattern composed of sources and corridors.

### 5.3. Policy Implications

Based on the results of previous modeling analysis, field study information, and opinions of local government departments and experts in the field of ecological landscape, policy recommendations are made at three levels: point, line, and surface, respectively.

(1) Comprehensive results in Table 4 and Table 6 and expert opinion on the river clip point, we make recommendations to establish a scientific pollution prevention and control research mechanism, monitoring on time, and real-time grasp of the water environment pollution dynamics. In view of the woodland pinch points, natural conservation sites and nature parks should be strengthened to maintain and protect the biodiversity of forest systems. In view of the regional problems of “barrier points”, we should establish a joint scheduling mechanism for the ecological water flow of important reservoirs and river sluice dams, vigorously implement wetland water diversion, and water replenishment and saline–alkali land management projects. Village joint construction in rural settlements and paddy fields should be promoted, and the intensive use of land should be improved.
(2) Analysis of the distribution of ecological corridors in Figure 7 shows that the potential ecological corridors are mainly distributed along the rivers in the study area, and the rivers are the key to linking the whole area. Ecological conservation corridors focus on wetland ecological protection. The ecological improvement area corridors highlight the construction of the Urban Wetland Park and Urban Dawen River Style Belt. The ecological corridor of ecological development areas focuses attention on ecological governance and the restoration of beach areas. Additionally, an ecological, modern, integrated, safe, and reliable road network traffic system should be established. Relying on the river embankment and national and provincial roads, an ecological road with a continuous ecological network should be further built, relying on the control project and county and township roads. Depending on the green belt controlled on both sides of the primary and secondary road network systems, a networked and diversified three-level slow traffic landscape greenway will be built.
(3) Based on model analysis and field research data statistics, the landscape ecological security pattern of the Dawen River basin was optimized and subdivided. For the PPZ, it is recommended to strengthen the ecological protection of wetlands, rivers, and lakes, and strive to improve vegetation coverage and enrich biodiversity. For the SPZ, it is recommended to install biological channels on both sides of important roads to maintain biological diversity. In addition, the development of geological relic resources should be strictly controlled, and the scale of the project should be controlled. For ERZ, it is recommended to focus on the protection of integrated tourism scenic spots and strengthen the protection of ecological quality and integrated management. For LPZ, the transformation from “extensive” to “ecological” should be accelerated, industrial ecological and ecological industrialization should be improved, and the ecological industry should be strengthened.

### 5.4. Limitations and Prospects 

In future research, the comprehensive observation index of natural resources can be enriched and the importance of water conservation, water, and soil conservation and other ecosystem services in Dawen River basin can be evaluated. The superposition of that research with the results of this paper will make the selection of ecological sources more comprehensive. In the construction process of the corridor, the appropriate width of the corridor can be further explored. In the process of barrier point identification, considering the high cost of removing barrier points, the minimum search radii, namely, an image dimensional size (30 m), can set different search radii to explore the impact of the threshold setting on landscape connected obstacle discrimination, and the accuracy of the research will be gradually improved to build a more optimized landscape ecological security pattern for the research region. Additionally, circuit theory is applicable to terrestrial species, so this paper limits the species to the study of terrestrial mammals in the Dawen River basin. The ecological security protection of non-land animals deserves further exploration.

## 6. Conclusions

Taking the Dawen River basin as the research area, this paper constructs the landscape ecological security pattern using the circuit principle. Considering the main 14 species of mammals in the region, the ecological corridors, including the key and potential corridors, were identified, and key points, including the pinch and barrier points, were identified. According to the field survey and relevant references, we analyzed the ecological problems in the Dawen River basin from the points, corridors, and areas, and constructed the landscape ecological security pattern.

Specifically, there are 128 ecological corridors in the Dawen River basin, with a total length of 1341.84 km, of which 83 are key corridors with a length of 95,321 km. The protection of ecological corridors is conducive to the migration of mammals. A total of 32 pinch points and 21 obstacle points were identified in this area. Among them, pinch points are the points with high probability during animal migration, which are concentrated in the east of the study area; barrier points are points in urgent need of ecological improvement and are scattered along the river basin. According to the degree of protection required, the necessity of regional ecological protection is divided into four types, and relevant ecological protection suggestions were put forward.

## Figures and Tables

**Table 1:** Summary of landscape pattern indexes.

Type	Name	Ecological Significance	Calculation Method
Quantitative index	NP	Describes the entire landscape heterogeneity, whose value size is positively associated with landscape fragmentation	NP=n
Fragmentation Index	DIVISION	Characterizes the degree of landscape fragmentation and the complexity of spatial structure reflecting human interference to some extent	$D=\frac{2\ln(\frac{P}{4})}{\ln(A)}$
Shape index	LSI	Reflects the plaque shape characteristics of the entire landscape	$V_{i}=\frac{D_{ij}}{A_{ij}}$
Cluster index	CONTAG	Reunion degree or extension trend of different patch types in the landscape	$CONTAG=\left[1+\frac{\sum_{i=1}^{m}\sum_{k=1}^{m}\left[\left(P_{i}\left(\frac{g_{ik}}{\sum_{k=1}^{m}g_{ik}}\right)\left[\ln(P_{i})\right]\left(\frac{g_{ik}}{\sum_{k=1}^{m}g_{ik}}\right)\right)\right]}{2\ln(m)}\right](100)$
Dispersion index	SPLIT	Describes the number of patches obtained when dividing the total region into parts of equal size	$S=\frac{A_{t}^{2}}{\sum_{n=1}^{n}A_{i}^{2}}$
Approximate index	SHDI	Reflects the degree of inuniformity in each patche on the landscape	$SHDI=-\sum_{i=1}^{k}P_{i}\log(p_{i})$

**Table 2:** Representative mammal species of Dawen River basin.

Order	Family	Latin Scientific Name	Alias
Rodentia	Cricetidae	Cricetulus barabensis	Chinese hamster
		Tscherskia triton	Cricetulus triton
	Muridae	Apodemus agrarius	Black-threaded rat
		Apodemus peninsulae	Mountain mouse
		Mus usculus	Mouselet
		Rattus norvegicus	Brown rat
Chiroptera	Rhinolophidae	Rhinolophus luctus	Hairy chrysanthemum bats
	Vespertilionidae	Pipistrellus pipistrellus	Bat
Erinaceomorpha	Erinaceidae	Erinaceus amurensis	Stabbs
Carnivora	Canidae	Canis lupus	Timber wolf
		Vulpes vulpes	Red Fox
	Mustelidae	Meles meles	Badger
		Mustela sibirica	Weasel
Lagomorpha	Leporidae	Lepus capensis	Rabbit

**Table 3:** The resistance indicators.

Resistance Factor	Weight	Index	Resistance Coefficient	
			Small Mammal	Large Mammal
a. Land cover	0.4	Cultivated land	100	65
		Forest	4	4
		Grassland	10	20
		Shrubland	2	1
		Wetland	1	2
		Water bodies	40	20
		Artificial Surfaces	500	600
		Bare Land	250	200
b. Slope/°	0.15	[0,8)	1	1
		[8,15)	20	10
		[15,25)	70	50
		[25,35)	120	75
		>35	200	120
c. Surface Undulation/m	0.15	[0,25)	2	1
		[25,50)	15	10
		[50,75)	75	50
		[75,100)	100	75
		>100	125	100
d. Distance from Grade I road/m	0.15	[0,200)	400	300
		[200,400)	320	240
		[400,800)	240	180
		[800,1600)	120	85
		≥1600	1	1
e. Distance from Grade II road/m	0.15	[0,150)	300	250
		[150,350)	240	200
		[350,750)	180	150
		[750,1200)	100	75
		≥1200	1	1

**Table 4:** The ecological protection zoning classification.

Zones	Scope Area	The Buffer	Land Use Type
PPZ	pinch points	1 km	Forest land, grassland, wetland, and water body
	ecological corridors	0.5 km	
	ecological sources	-	
ERZ	obstacle points	1 km	Farmland and village
	areas with IS > 10%	-	
SPZ	potential ecological corridor	0.5 km	Forest land, grassland, wetland, and water body
	ecological source	2 km	
LPZ	other spaces excluding above three types	-	others

**Table 5:** Distribution of pinch points and obstacle points in the Dawen River basin.

No.	Name	Pinch Points	Barrier Points
1	DY	13	7
2	DP	2	5
3	FC	2	2
4	LS	4	2
5	NY	1	2
6	TS	2	1
7	WS	2	-
8	XT	6	2

**Table 6:** Statistical table of zoning situation and area for all districts and cities.

Area	PPZ	SPZ	ERZ	LPZ	Total
DY	13,835.83	64,065.43	3303.27	94,228.31	175,432.84
DP	14,244.15	27,920.01	1729.17	89,819.68	133,713.01
FC	1421.18	16,032.73	427.59	108,759.01	126,640.50
LS	738.11	12,092.93	515.79	82,715.38	96,062.21
NY	1762.74	12,463.96	674.23	97,367.86	112,268.79
TS	2903.22	9134.90	362.50	21,530.22	33,930.84
WS	649.97	5738.91	119.88	82,316.01	88,824.76
XT	6620.62	53,605.73	2031.82	130,771.53	193,029.70
Total	42,175.81	201,054.59	9164.25	707,508.00	959,902.65

**Table 7:** Key areas for protection and restoration in the Dawen River basin.

	Status Type	Region	Existing Problem
Pinch points to be protected	Rivers	DP	Industrial development is extensive, low-quality, and inefficient, and the ecological overdraft problem is serious. Affected by water conservancy project coercion and human agricultural activities, the water conservation capacity has decreased. The coastal pollution discharge problem is serious, and rivers gradually shrink and narrow.
		FC	
		DY	
		TS	
	Woodland Construction Land	TS	Falling forest coverage; forest network fragmentation has increased. Lower biodiversity, putting pressure on the ecological environment and reducing connectivity between habitats.
		XT	
		FC	
Barrier points to be		XT	Changing the original flow direction and speed of water bodies can partially hinder the migration path of aquatic and terrestrial organisms. Ecological community structure has received obvious influence, decreased original river regulation and storage capacity, and frequent flood disasters.
addressed		FC	
		DP	
		TS	
	Road land	DY	During the development, protective forests, wetlands, and woodlands are damaged and isolated on both sides of the highway.
		TS	
	Rural settlements and paddy fields	DP	Intensive human activities; the use of modern agricultural technology gradually rice farming from mixed planting to single planting, and the destruction of habitat connectivity in some mechanized planting areas.
		FC	
		XT	
		DY	

## Data Availability

The data are proprietary or confidential in nature and may only be provided with restrictions. The data presented in this study are available on request from the corresponding author.
